# Breast microecology improvement using probiotics following needle aspiration in patients with lactational breast abscess: a multi-center randomized double-blind controlled trial

**DOI:** 10.1038/s41598-022-20756-w

**Published:** 2022-10-06

**Authors:** Yi Zhang, Yajun Gao, Jing Qin, Xiaoting Li, Fei Jiang, Yuanxuan Cai, Hui Feng, Xidong Gu, Mingze Gao, Lijuan Wang, Yiqi Lin, Yingyi Fan, Bucun Xu, Enli Wang, Qing Shao

**Affiliations:** 1 Center for Prevention and Cure of Breast Diseases, Maternal and Child Health Hospital, Haidian District, Beijing, 100080 China; 2 Key Laboratory of Carcinogenesis and Translational Research (Ministry of Education/Beijing), Department of Radiology, Peking University Cancer Hospital and Institute, Beijing, China; 3 Breast Department, Obstetrics and Gynecology Hospital, Kaifeng, Henan Province China; 4 Breast Department, Guangzhou Women and Children’s Medical Center, Guanzhou, Guangdong Province China; 5 Breast Department, Taiyuan Maternity and Child Health Care Hospital, Taiyuan, Jiangxi Province China; 6 Breast Department, Zhejiang Provincial Hospital of Chinese Medicine, Hangzhou, Zhejiang Province China; 7 Breast Department, Changde First Hospital of Traditional Chinese Medicine, Changde, Hunan Province China; 8 Breast Department, Sanya City Womenfolk and Infant Health Care Hospital, Sanya, Hainan Province China; 9 Breast Department, Jilin Women and Children Health Hospital, Changchun, Jilin Province China; 10 Breast Department, Beijing University of Chinese Medicine Third Affiliated Hospital, Beijing, China; 11 Breast Department, Anyang Maternal and Child Health Care Hospital, Anyang, Henan Province China; 12 Breast Department, Shenzhen Maternity and Child Health Care Hospital, Shenzhen, Guangdong Province China; 13 Breast Department, Jiangyin People’s Hospital, Jiangyin, Jiangsu Province China

**Keywords:** Microbiology, Diseases, Health care, Medical research

## Abstract

Although oral probiotics can improve breast microecology and alleviate the inflammatory response, there are no data regarding cases with existing abscesses. We aimed to investigate the effect of *Lactobacillus fermentum* CECT5716 during needle aspiration in patients with lactational breast abscesses. Patients (aged 20–41 years) with lactational single-cavity breast abscesses (diameter 3–6 cm) from 12 hospitals were randomly assigned to the experimental (n = 51) and control groups (n = 50). Outcome measures included the abscess cure rate on treatment day-5, delactation rate, relieving pain rate, and number of needle aspirations until day-28. The experimental group’s 5-day cure rate (43.1%) was significantly higher (*p* < 0.05). Breastfeeding continuation on day-5 did not differ significantly (experimental group: 88.2%, control group: 96.0%, *p* = 0.269). In the experimental and control groups, 19.6% and 14.0% of patients experienced moderate to severe pain on day-5, respectively, with no statistically significant differences (*p* = 0.451). Four patients in each group developed diarrhea, with adverse reaction rates of 7.84% and 8.0%, respectively. No adverse reactions were reported in the infants. *L. fermentum* can shorten the healing time in patients with lactational breast abscesses.

*Trial registration* This study was registered in the Chinese Clinical Trial Registry (http://www.chictr.org.cn), registration number: ChiCTR2000032682, registration date: 6/May/ 2020; first entry date: 11/May/2020.

## Introduction

The World Health Organization (WHO) maintains that breastfeeding benefits both the mother and the child^1,2^. These benefits include aiding in the protection of the child against a variety of acute and chronic diseases. A review of studies conducted in developing countries has revealed that the risk of death in non-breastfed infants is 6 to 10 times higher than that in breastfed infants during the first few months of life^3,4^. In China, poor breastfeeding and complementary feeding practices are widespread, thereby impairing the success of breastfeeding.

Lactational mastitis and its associated breastfeeding-related problems are key factors that represent the first medical cause for undesired weaning^5^. Furthermore, once the inflammation progresses and develops into an abscess, treating the condition would necessitate needle aspiration or even incision and drainage, which may reduce the breastfeeding rate. At present, the problem of drug resistance caused by antibiotic abuse is increasingly common; hence, it is imperative to explore new methods to treat an infection. Probiotics, which serve as a prospective treatment option, are safe and do not have a drug resistance problem. Studies show that oral probiotics can improve the breast microecology and thus alleviate the inflammatory response^6^; however, there is a lack of experimental data corresponding to cases with existing abscesses.

To verify the effect of *Lactobacillus fermentum* CECT5716 in the treatment of patients with lactational breast abscesses, we aimed to conduct a randomized controlled double-blind trial across 12 hospitals in China between May 2020 and August 2020. The primary outcome measure was the abscess cure rate on day-5 of the treatment. The secondary purpose were the comparison of the breastfeeding rate during the treatment period, Pain rating on day-5 of treatment and Adverse reactions.

## Methods

### Study design and setting

A prospective, multicenter, randomized, double-blind, controlled clinical trial was conducted, which involved the participation of 12 centers and aimed verifying the effect of *L. fermentum* CECT5716 in the treatment of patients with lactational breast abscesses. The 12 selected hospitals were from 11 different municipalities and prefecture-level cities in China, and the distribution was relatively uniform. Random allocation was performed using a centralized randomization system (Zhejiang Taimei Technology Co., Ltd.). The probiotics and placebo were blinded as Product A or Product B; they showed no difference in appearance, and the product manager, on obtaining the random group number from the central random system, distributed Product A or Product B to the patients. For allocation of the participants, a list of random numbers, generated from an interactive web response system (eBalance 5.X), was used. Participants were randomly assigned to one of the two treatment groups with a 1:1 ratio. The block randomization procedures were implemented using random block sizes of 4.eBalance 5.X, which kept the allocation information of all participants, including their treatment groups, concealed until the clinical trial was completed and trial unblinding was required. Randomized patients received all products from the product manager during the study period, according to the intervention they were allocated; Replace empty bags with new products at each follow-up visit. Study investigators, research coordinators, and the patients were blinded to the treatment allocation. The collected data were uploaded for review by the doctor to the Golden Data System (Xian Shu Ju Ru Jin tech co. ltd, Xian, China.). The doctors decided whether to perform a new puncture based on the results of the ultrasound.

### Ethics approval and consent to participate

This study was approved by the Medical Ethics Committee of the Xiyuan Hospital of the China Academy of Chinese Medical Sciences (2020XLA025-1). After screening suitable cases, trained doctors spoke to the patients and obtained informed consent.

### Selection of participants

The following inclusion criteria were used in selecting participants: (a) patients aged between 20 and 45 years who were diagnosed with a lactational breast abscess; (b) patients who presented with a single-cavity unilateral breast abscess with a maximum diameter that was ≥ 3 cm and ≤ 6 cm as measured on the ultrasound, who did not exhibit epidermal ulceration, and who had undergone needle aspiration; and (c) patients who agreed to participate in the study and provided informed consent in writing.

The following exclusion criteria were applied: (a) patients who had previously presented with a breast abscess during the current pregnancy and lactation period; (b) patients in whom a medical examination revealed comorbid infections of other organs, such as puerperal infection; (c) patients with severe comorbid organ dysfunction (e.g., diabetes and hepatic, renal, and immune insufficiency); (d) patients who did not maintain milk production in the affected breast through breastfeeding, breast pumping, or manual expression; and (e) patients whose body temperature was > 37.5 °C within 24 h of testing.

The discontinuation criteria were as follows: (a) Patients who presented with severe diarrhea (watery stool > 5 times/24 h) during the treatment process. These patients were treated as discontinued cases and were promptly reported to the ethics committee; further, if the patient’s body temperature was ≥ 37.5 °C, their needle aspiration frequency was adjusted, they were guided in terms of lactation methods, or they were administered an active antibiotic treatment for 72 h. Even then, patients whose body temperature remained abnormal were withdrawn from the study. (b) Participants with poor compliance or who consumed the specified prohibited drugs mentioned in the study protocol during the experiment. (c) Participants who experienced other serious adverse events, such as cancer, human immunodeficiency virus(HIV), etc.

**Figure 1:**
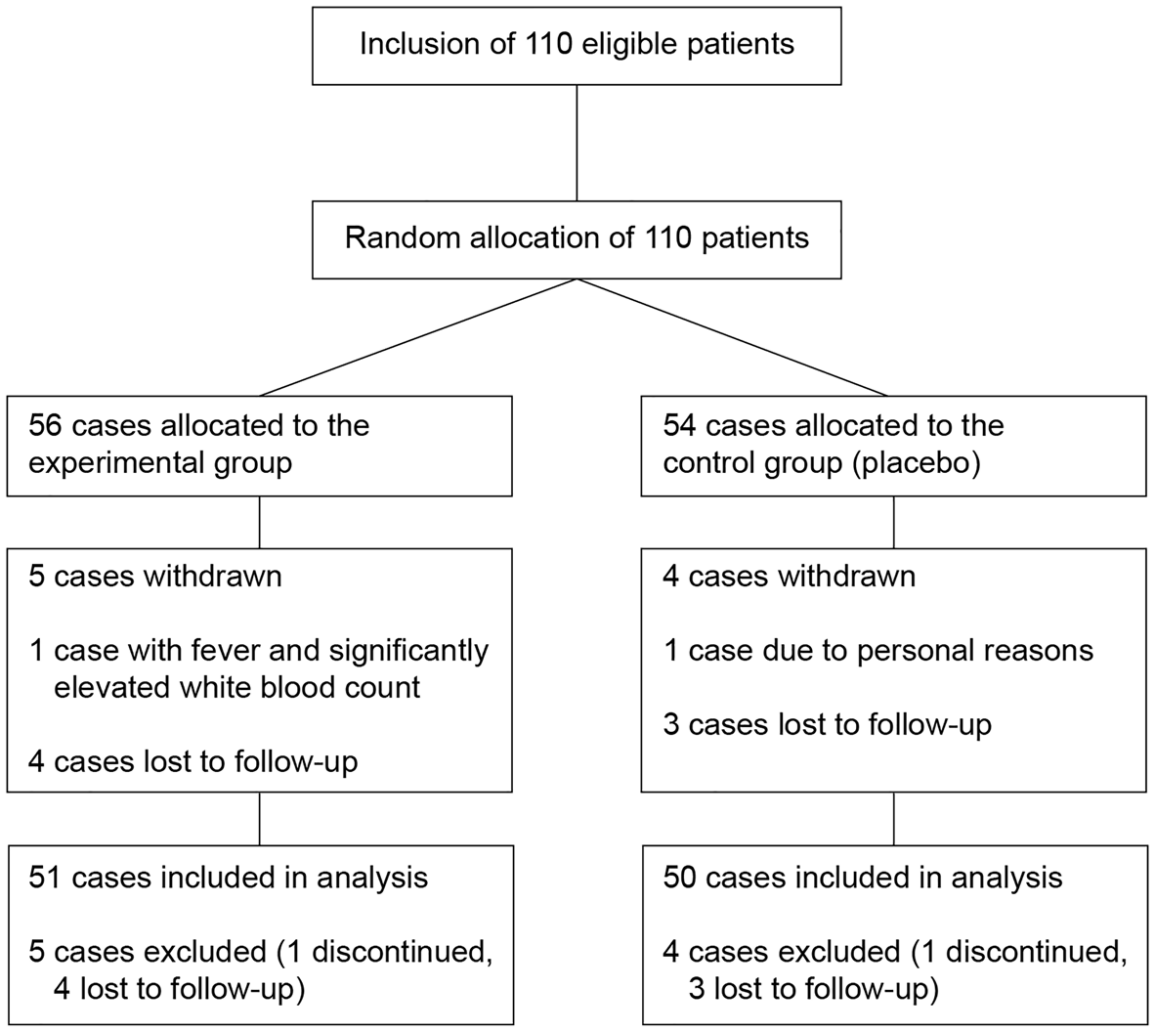
Patient selection flow chart.

Based on the criteria above, a total of 110 patients were enrolled in the clinical trial between May and August 2020, of whom 101 completed the trial (Fig. 1).

Before the start of the study, unified training was provided in all centers, and cases were registered in all centers until recruitment was completed. The research commenced in May 2020 and was completed in August 2020. After screening suitable cases, trained doctors spoke to the patients and obtained informed consent. In addition, each patient received 100 RMB for each review and up to 800 Renminbi(RMB) for transportation.

### Interventions

Both patient groups underwent routine needle aspiration of the breast abscess. Patients who met the inclusion criteria provided written informed consent prior to the needle aspiration. Complete routine blood tests and ultrasound examinations were performed before the operation. The most suitable puncture point (generally near the edge of the abscess cavity but far from the areola) and puncture path were then determined based on the location of the lesion; subsequently, the sites were routinely disinfected and draped and were subjected to infiltrative anesthesia with 1% lidocaine hydrochloride. Under the full guidance of an ultrasound, the needle was inserted into the fluid-filled hypoechoic area of the breast abscess, and the pus was aspirated and sent for bacterial culture. The puncture site was covered with sterile tape or gauze. In addition to needle aspiration treatment, the experimental group took one sachet of *L. fermentum* CECT5716 in warm water 30 min after the main meal once a day for 4 weeks continuously from the first needle aspiration treatment session. *L. fermentum* CECT5716 (Hereditum LC40) was manufactured by Biosearch Life (Granada, Spain). The participants continued to breastfeed during the testing period, and the number of needle aspirations was adjusted as needed. The control group took maltodextrin as a placebo in the same manner. Medical staff set up a professional follow-up team to record the main observation indicators of patients through outpatient or wechat. The patients were visited on day 1, day 3, day 5, and day 7 10, day 15, day 21 and day 28, and the outpatient follow-up was arranged according to the patient's Symptoms after the punctures.

During the 28 days of the study, the size of the abscess cavity was measured using the ultrasound on days 1, 5, 15, and 28, and the recovery, pain index, lactation, and maternal and infant safety were evaluated by the physician. The collected data were immediately uploaded by the doctor on the Golden Data System. After complete enrollment and follow-up, data were derived from the system. At the same time, the central random system was unblinded and the data were handed over to the statistical experts for processing. Each case was verified to ensure its authenticity. The clinical research associate monitored the integrity and correctness of the data through the Golden Data System.

This study was approved by the Medical Ethics Committee of the Xiyuan Hospital of the China Academy of Chinese Medical Sciences (2020XLA025-1) and was registered in the Chinese Clinical Trial Registry (registration number: ChiCTR2000032682, registration date: 06/05/2020; first entry date: 11/May /2020). Research was performed in accordance with relevant guidelines and regulations.

### Outcomes/measurements

The criteria for a cured breast abscess were as follows: the complete disappearance of the abscess; no local redness, swelling, heat, or pain; and normal body temperature.

Pain rating: A visual analog scale was used to analyze and compare the degree of pain perceived by the two groups during the last milk removal session; pain scores of 0–3 points indicated mild pain, while those of 4–7 points and above indicated moderate to severe pain.

#### Estimation of the sample size

According to relevant data derived from literature reports and clinical experience statistics^7^, it was preliminarily estimated that under the current routine treatment plan, the complete cure rate on day-5 would be 41.5%, while pilot testing indicated that when the treatment was combined with a breast microecological intervention, the cure rate would increase to 70%. Based on the calculations performed using PASS15.0 (HyLown Consulting LLC, Atlanta, GA, USA), the minimum sample size needed per group was found to be 44 patients, and if the proportion of patients that were lost to follow-up or were withdrawn was kept within 20%, then 55 cases would be needed in each group, thereby a total of 110 cases would be needed across both groups.

### Analysis

Data processing and statistical analyses were performed using SPSS 23.0 (IBM Corp., Armonk, NY, USA). All the tests were two-tailed and the significance level was α = 0.05. Measurement data were either described using means/standard deviations or medians/interquartile ranges depending on their distribution; the between-group comparison was performed using a t-test or non-parametric test. Count data were described using frequencies and percentages, and the between-group comparison was performed using the chi-squared test or Fisher’s exact test. Logistic regression analysis was used to estimate the odds ratio (OR) and 95% confidence interval (CI) of the primary outcome measure between the two groups and to analyze the influencing factors, with the inclusion of clinically important variables using the forced entry method. The analysis set was the “full analysis set,” which is based on the intention-to-treat principle; all the randomized (eligible) participants were included in the analysis, and patients with missing data associated with the primary outcome measure or those without any follow-up data were excluded.

## Results

### Characteristics of the study subjects

Between May and August 2020, a total of 110 patients from 12 centers were randomized for participation in this clinical trial, among whom, 101 patients completed the trial and were included in the analysis, and nine cases dropped out (no primary outcome measure) and were excluded from the analysis. The experimental group included 51 patients, with a median age of 31 years (20–39 years), and the control group included 50 patients, with a median age of 30 years (20–41 years). The other basic information is summarized in Table 1.

**Table 1:** Basic information of the two groups.

Variable	Value	Experimental group		Control group	
		N	%	N	%
Pregnancy status	Singleton	42	82.4	41	82.0
	Twins	9	17.6	9	18.0
Delivery status	Normal delivery	35	68.6	36	72.0
	Cesarean section	16	31.4	14	28.0
Breastfeeding status	Breastfeeding	32	62.7	30	60.0
	Mixed feeding	15	29.4	17	34.0
	Others	4	7.8	3	6.0
*Treatment history	Yes	22	43.1	21	42.0
	No	29	56.9	29	58.0
Maximum diameter of abscess cavity (cm)	3 ≤ n < 4	16	31.4	19	38.0
	4 ≤ n < 5	17	33.3	13	26.0
	5 ≤ n ≤ 6	18	35.3	18	36.0

*Treatment history included the use of antibiotics before and during the study period. Of the 22 cases in the experimental group, 18 were treated with cephalosporins and four with other antibiotics. Of the 21 cases in the control group, 14 were treated with cephalosporins and seven with other antibiotics.

### Primary outcome measure: comparison of the cut-off abscess cure rate on day-5 of treatment

#### Univariate analysis

As shown in Table 2, the cut-off abscess cure rate on day-5 of treatment in the experimental group was 43.1%, while that in the control group was 18.0%. The difference in the cure rate between the two groups was 25.1%, which was statistically significant (Pearson’s chi-squared = 7.500, *p* = 0.006).

**Table 2:** Cut-off abscess cure rate on day-5 of treatment in the two groups.

	Experimental group		Control group		Pearson’s chi-squared	*p*
	N	%	N	%		
Cure/remission	22	43.1	9	18.0	7.500	0.006
Not cured	29	56.9	41	82.0		

#### Multivariate analysis

A logistic regression analysis was performed to analyze the factors influencing the cut-off abscess cure rate on day-5 of treatment, and the forced entry method was used to include variables, such as pregnancy status, breastfeeding status, and treatment history. The results are presented in Table 3. After adjustments for the variables, such as pregnancy status, breastfeeding status, and treatment history, the treatment group was still an influencing factor on the cut-off abscess cure rate on day-5 of treatment. The risk of not being cured in the experimental group was 0.282 compared with that in the control group, and the difference was statistically significant [Odds ratio (OR) OR 0.282, 95% CI 0.112–0.713, *p* = 0.007)].

**Table 3:** Multivariate logistic regression analysis of the factors influencing the cure rate on day-5.

Factor	OR	95% CI	*p*
**Group**			0.007
Experimental group	0.282	0.112–0.713	
Control group	1	–	
**Pregnancy status**			0.711
Singleton	1	–	
Twins	0.805	0.255–2.537	
**Breastfeeding status**			0.202
Breastfeeding	1	–	
Mixed feeding	0.468	0.166–1.317	0.150
Others	0.240	0.026–2.236	0.210
**Treatment history**			0.852
Yes	1.091	0.438–2.719	
No	1		

CI, confidence interval; OR, odds ratio.

### Between-group comparison of the breastfeeding rate during the treatment period

The proportion of patients who continued breastfeeding on the affected breast on day-5 of treatment was 88.2% and 96.0% in the experimental and control group, respectively. The difference in the cure rate between the two groups was 7.8%, which was not statistically significant (Fisher’s exact probability *p* = 0.269). See Table 4 for details.

**Table 4:** Between-group comparison of the breastfeeding rate on day-5 of treatment.

	Experimental group		Control group		*Fisher' p*
	N	%	N	%	
Continued breastfeeding	45	88.2	48	96.0	0.269
Stopped breastfeeding	6	11.8	2	4.0	

### Pain rating on day-5 of treatment

As shown in Table 5, the proportion of patients with moderate to severe pain on day-5 of treatment was 19.6% in the experimental group and 14.0% in the control group. The difference in the cure rate between the two groups was 5.6%, which was not statistically significant (Pearson’s chi-squared = 0.567, *p* = 0.451).

**Table 5:** Between-group comparison of the pain rating on day-5 of treatment.

	Experimental group		Control group		Pearson’s chi-squared	*P*
	N	%	N	%		
Mild	41	80.4	43	86.0	0.567	0.451
Moderate to severe	10	19.6	7	14.0		

### Adverse reactions

After the treatment, four patients in the experimental group developed diarrhea and adverse reaction rate was 7.84% (4/51); further, four patients in the control group developed diarrhea, and the adverse reaction rate was 8.0% (4/50). The affected patients in both the groups recovered spontaneously in the short-term without a medication-based intervention. Thus, we believe that this strain does not affect the normal gut microbiota of the human body. During the trial, no adverse reactions were reported in the infants of the participants.

## Discussion

In the present study, a total of 101 women were selected as participants from across the country, and the outcomes of the therapeutic effects of *L. fermentum* were similar to the results of the studies on mastitis from other countries. Our findings indicated that the 5-day cure rate in the experimental group was 43.1%, which was significantly higher than that of the control group (18.0%), thus implying the substantial effect of the strain in the abscess cure time reduction. The selection of the appropriate cure time was based on the integration of results of other studies^8^, which indicated a mean disease duration of 8 days. Given the possibility of generating positive results, the cure rate on day-5 was selected as the primary outcome measure. In terms of the continued breastfeeding rate, there were no statistically significant differences between the two groups. This may have been because all the patients had single-cavity abscesses that were easier to treat clinically and had a relatively small mental and physical effect on the patients. However, the long-term effects of these abscesses on breastfeeding are still not known; thus, long-term observation and follow-up periods are still required. The pain rating depends on the subjective evaluations of the clinicians and participants, which involve a number of influencing factors, such as family environment, breastfeeding habits, etc. Therefore, these variables did not show significant differences. There was also no difference in the number of needle aspirations during the abscess treatment between the two groups, which may have been influenced by the different technical standards of the multiple centers, as well as by the different types of bacterial infections, previous use of antibiotics, and use of treatment methods such as Chinese medicine.

Ensuring an adequate nutritional supply during infancy and early childhood plays a vital role in assuring the normal growth and development of children. In a study conducted by the WHO in 2006, it was reported that among 9.5 million children, malnutrition directly or indirectly contributed to approximately one-third of the deaths in children < 5 years of age^1,9^. In addition, malnutrition during the first 2 years of life can result in long-term growth and health impairment. Jones et al.^10^ demonstrated that optimal breastfeeding could reduce the global mortality rate among children aged < 5 years by 13%. In 2002, the WHO and United Nations Children's Fund released the “Global strategy for infant and young child feeding” guidelines^11^ that recommend exclusively breastfeeding a child until 6 months, followed by complementary feeding until 2 years of age and beyond. However, in reality, the global breastfeeding rate is a cause for concern. Arabi et al. surveyed 28 developing countries and found that only 25% of 0- to 5-month-old infants were exclusively breastfed^12^. In China, poor breastfeeding and complementary feeding practices are widespread. A prospective study conducted in 2014 revealed that 3–20% of breastfeeding women had acute mastitis during the feeding process^13^. Pevzner et al.^14^ demonstrated that lactational mastitis could contribute to the premature cessation of breastfeeding. Therefore, the safe and effective treatment of lactational mastitis and shortening of the treatment time are currently crucial issues that need to be resolved.

Cullinane et al.^15^ verified that the presence of *Staphylococcus aureus* in milk increased the risk of developing mastitis. *S. aureus* is the predominant pathogen in postpartum breast abscesses, accounting for 32–95% of all culture-confirmed cases, and Methicillin resistance was higher than 60%^16,17^. Peripartum antibiotherapy has emerged as a strong risk factor for human mastitis due to the selection of antibiotic-resistant staphylococci in the mammary environment and the elimination of potential natural competitors^18^. It was previously believed that breast milk itself is sterile and that the contained bacteria originate from the infant’s mouth^19^, entering the mother’s milk retrogradely because of the infant’s sucking action during the breastfeeding process. Owing to the continuous advancement in research methods, more than 800 species of bacteria have been detected in breast milk with the application of metagenomics, which suggests that mastitis is not simply due to bacterial invasion and infection but is the result of changes in breast microecology, which could destroy the bacterial diversity^20^. Benno et al.^21^ showed that anaerobic bacteria that cannot survive in aerobic environments could be detected in the breast milk, thus demonstrating that the bacteria in breast milk do not entirely originate from the external world. Recently, the presence of an entero-mammary pathway has been hypothesized^22^, suggesting that the bacteria found in breast milk could have entered the mammary glands via an endogenous route from the mother’s gastrointestinal tract or vagina, resulting in their secretion with the milk. The milk and bacteria together form a dynamically balanced microenvironment. The disruption of this balance can lead to localized or diffuse infections and can result in lactational mastitis^23^. These new discoveries presented us with the possibility of treating mastitis through the regulation of the microbiota.

Once lactational mastitis progresses into the development of a breast abscess, it becomes necessary to drain the pus to enhance the body’s resistance to pathogens and viruses. However, the traditional incision and drainage approach gives rise to certain problems such as a large incision and painful dressing changes, which can cause substantial agony in patients and psychological trauma due to the resulting effects on their physical appearance. In recent years, ultrasound-guided needle aspiration of abscesses has gradually been widely accepted in clinical practice. Compared with traditional incision and drainage techniques, the advantages of the latter include a small incision, a high cure rate, fewer complications, and better preservation of breast aesthetics^8^. Ding et al.^7^ found that the factors that are associated with the abscess itself, including the amount of pus collected during the first puncture, abscess location, and abscess size, are key to the recovery process following needle aspiration treatment. Further investigation is needed to determine other factors that affect the postoperative recovery of patients following the needle aspiration of a breast abscess.

Spanish scholars have isolated *L. fermentum* CECT5716 from healthy breast milk and found that this type of breast milk probiotic can effectively antagonize pathogenic bacteria, alleviate symptoms of swelling and pain during breastfeeding, and enhance both maternal and child immunity and gut health. To investigate this, Hurtado et al.^24^ randomly divided 291 women who were in the puerperal period into an experimental group that received one probiotic capsule (*L. fermentum* CECT5716) a day and a control group that received one maltodextrin placebo capsule a day for a 16-week continuous intervention period. Their results showed that following the probiotic intervention, the incidence of mastitis was reduced by 51%. Arroyo et al.^25^ conducted a 21-day study on 352 women with lactational mastitis who were randomly assigned to three groups. Two groups were given 9 log10 colony-forming units (CFU) L. fermentum CECT5716 and L. salivarius CECT5713, respectively, while the control group was treated with routine antibiotics. The results showed that at day 21, the number of colonies in the probiotics group (2.61 and 2.33 log10 CFU/mL) was significantly lower than that in the antibiotics group (3.28 log10 CFU/mL), and the women in the probiotics group had significantly better pain relief than those in the antibiotics group. The recurrence rate in the antibiotic group (30.7%) was significantly higher than that in the probiotics group (χ^2^ = 27.08, P&L;0.001). The mechanism of action of *L. fermentum* could involve colonizing the digestive tract after entering the body via oral administration, reaching the breast tissue via the endogenous entero-mammary pathway, engaging in the competitive inhibition of pathogenic bacteria, and optimizing breast microecology, thereby resulting in the alleviation of the inflammatory response in the breast^6^. Furthermore, the recurrence and delactation rates in the antibiotic group were both higher than those in the probiotic group, which is associated with the destruction of the normal human microecology by common broad-spectrum antibiotics. This study has certain limitations, including the small sample size and multicenter treatment administration that may have affected the results due to the different treatment techniques in the different centers. Therefore, the comparison of puncture times was of little significance and was not counted. Family support, culture, and mastery of lactation techniques all have an impact on breastfeeding pain and early weaning. Therefore, only the cure rate on day 5 showed an advantage in this study, and a larger sample study is needed to further clarify.

## Conclusion

In summary, for patients with a single-cavity lactational breast abscess with a maximum diameter of 3–6 cm, the oral administration of *L. fermentum* CECT5716 during needle aspiration treatment can shorten the healing time and has no effect on safety during breastfeeding.

## Data Availability

All data generated or analyzed during this study are included in this published article.
